# Plastic Instability in Medium-Carbon Tempered Martensite Steel

**DOI:** 10.3390/ma14164609

**Published:** 2021-08-17

**Authors:** Hai Qiu, Rintaro Ueji, Tadanobu Inoue, Yuuji Kimura

**Affiliations:** Research Center for Structural Materials, National Institute for Materials Science, 1-2-1 Sengen, Tsukuba 305-0047, Ibaraki, Japan; Ueji.Rintaro@nims.go.jp (R.U.); Inoue.Tadanobu@nims.go.jp (T.I.); Kimura.Yuuji@nims.go.jp (Y.K.)

**Keywords:** Lüders deformation, heterogeneous plastic deformation, medium-carbon steel, plastic instability, digital image correlation

## Abstract

Inhomogeneous plastic deformation damages the surface quality of a product in the metal forming process. Therefore, it is necessary to investigate the plastic instability of a metal. Tempered martensite is a common microstructure of medium-carbon steel. Plastic instability (Lüders phenomenon, Portevin-Le Châtelier phenomenon) in this phase was investigated by a uniaxial tension test performed at room temperature. The formation and propagation of a plastic band were analyzed via two-dimensional digital image correlation, and the strain and strain-rate fields were experimentally evaluated. The results obtained are as follows: (1) there was no clear yield plateau on the stress–strain curve; (2) Lüders phenomenon was present, but the Portevin-Le Châtelier phenomenon was not found; (3) in the Lüders deformation process, local strain distribution in tempered martensite is more complicated than that in ferrite.

## 1. Introduction

Plastic instability occurs in the deformation process of some crystalline materials in the form of single or multiple plastic bands. Piobert [1] and Lüders [2] first reported that plastic instability took place when mild steel transited from an elastic to a plastic state. Portevin and Le Châtelier [3] found that plastic localization existed in a certain range of a plastic deformation process in aluminum-based alloys and low-carbon steels. Their results showed that plastic instability could take place not only in the elastic-to-plastic transition region but also during the process of plastic deformation. The plastic instability occurring in the former period is referred to as the Lüders phenomenon, and that in the latter period is the Portevin-Le Châtelier (PLC) phenomenon. Some materials have only one of the two types of plastic instabilities [4,5], and some have both [6]. The type of plastic instability can be identified from the typical characteristic on the tensile curve: a yield plateau for the Lüders deformation [4,5] or a jerky flow (a series of serrations) for the PLC effect [7,8,9,10,11]. Lüders deformation is dependent on the applied stress [12,13], grain size [14,15], strain rate [16,17,18], specimen size [18,19], and temperature [20]. The PLC effect is strongly influenced by the temperature [21] and strain rate [22].

Although the micro-mechanisms of the Lüders and PLC phenomena have been widely studied, they have not been clearly explained nor confirmed. Cottrell [23,24] first proposed a dislocation model that assumes that the two types of plastic instability are related to the interactions between solute interstitials, such as C and N, and mobile dislocations. The dislocations are initially locked by solute interstitials, which tend to form Cottrell atmospheres around them. The pinning of dislocations is associated with the increase in yield strength (i.e., hardening). When the stress threshold for unlocking or multiplying these dislocations is exceeded, the dislocations are unpinned, and a rapid multiplication of mobile dislocations occurs; as a result, yield strength decreases (i.e., softening). The pinning and the unpinning of dislocations result in strain aging [25]. It is generally believed that the Lüders phenomenon is caused by static strain aging (SSA) [26,27], and the PLC effect is attributed to dynamic strain aging (DSA) [10,28,29,30]. Onodera et al. found that the Cottrell atmosphere did not agree with the Lüders deformation in an alloy, Al–4Cu–0.5Mg–0.5Mn [31]. Hahn [32] proposed another model in which the dominant mechanism of Lüders band formation is attributed to rapid dislocation multiplication.

Steel has several elementary microstructures, such as ferrite, austenite, bainite, martensite, and pearlite. The Lüders phenomenon is known to occur in the ferrite [15,16] or austenite [33] phase, and the strain-induced phase transformation from metastable austenite to martensite leads to the PLC effect [26,34]. Due to the presence of ferrite or austenite, plastic instability can also take place in multi-phase steels containing either phase, e.g., ferrite/pearlite steel [4,5], ferrite/austenite steel [2,6], and ferrite/austenite/martensite steel [34]. However, the possible occurrence of plastic instability in phases other than ferrite and austenite was not reported in the literature.

Tempered martensite, which has an excellent balance of strength and toughness, is the most common microstructure used in medium-carbon steel. However, plastic instability in this phase is unknown. In this study, the evolution of strain and strain rate in the tempered martensite of medium-carbon steel in a tension test was analyzed via digital image correlation (DIC), and the possibility of the occurrence of plastic instability was investigated by the obtained experimental results.

## 2. Experimental Methods

Hot-rolled steel plates 120 mm long × 60 mm wide × 1.2 mm thick were used as as-received material whose chemical composition is 0.3 C, 1.5 Mn, and the balance Fe (in wt%). The as-received plates were heat-treated to obtain the desired microstructures in the present study:

(1) Tempered martensite:
  Tempered martensite is the microstructure in focus for the present study. The as-received plates were heated at 850 °C for 5 min, and then were water quenched to produce martensite. The water-quenched plates were heated in a furnace (600 °C) for 20 min, followed by furnace cooling to room temperature. The tempered martensite steel plates were denoted as QT steel, which is composed of fully tempered martensite.
(2) Ferrite:
  The Lüders phenomenon in ferrite was well investigated in the literature [2,4,5]. We selected ferritic steel as a reference for comparing the characteristic of plastic instability between the tempered martensite steel and ferritic steel. The as-received steel plates were heated at 850 °C for 5 min, and then the furnace cooled to room temperature. At 850 °C, the microstructure of the as-received steel transformed into austenite. The austenite transformed into ferrite and pearlite during furnace cooling, and the volume fraction of the ferrite and pearlite was 95.4% and 4.6%, respectively. The obtained plates were denoted as F steel.

**Figure 1:**
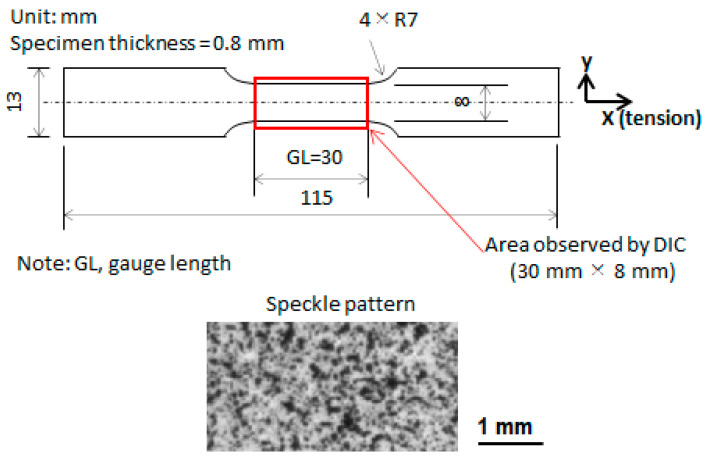
The size of the dog-bone-type specimen and the spackle patterns on the front surface of the specimen.

Dog-bone-type specimens (c.f. Figure 1) with a parallel part 30 mm long, 8 mm wide, and 0.8 mm thick were machined from QT steel (two specimens, and their numbers: QT-1 and QT-2) and F steel (two specimens, and their numbers: F-1 and F-2). Their front surfaces were sprayed with white and black paint to make speckles for DIC analysis. An extensometer with a gauge length (GL) of 30 mm (equal to the length of the parallel part of the specimen) was attached to the back surface. Tension tests were performed on the four specimens at room temperature and at a crosshead speed of 0.01 mm/s. The deformation process on the front surface was recorded successively at a time interval of 0.5 s using a camera. The digital images (area: 30 mm × 8 mm) obtained were processed using VIC-2D software with a subset size of 9 pixels × 9 pixels (246 μm × 246 μm) and a step of 5 pixels (137 μm) to produce the displacement field, strain field, and strain-rate field. In the DIC operation, the displacement uncertainty is 0.02 pixels. The DIC measurement area covers the whole GL. In the present study, the two results were obtained from tension tests: (1) macroscopic stress–strain curves showing the global image of the tensile property and (2) the evolution of plastic deformation in terms of the strain field and strain-rate field.

## 3. Results and Discussion

### 3.1. Macroscopic Stress–Strain Curves

**Figure 2:**
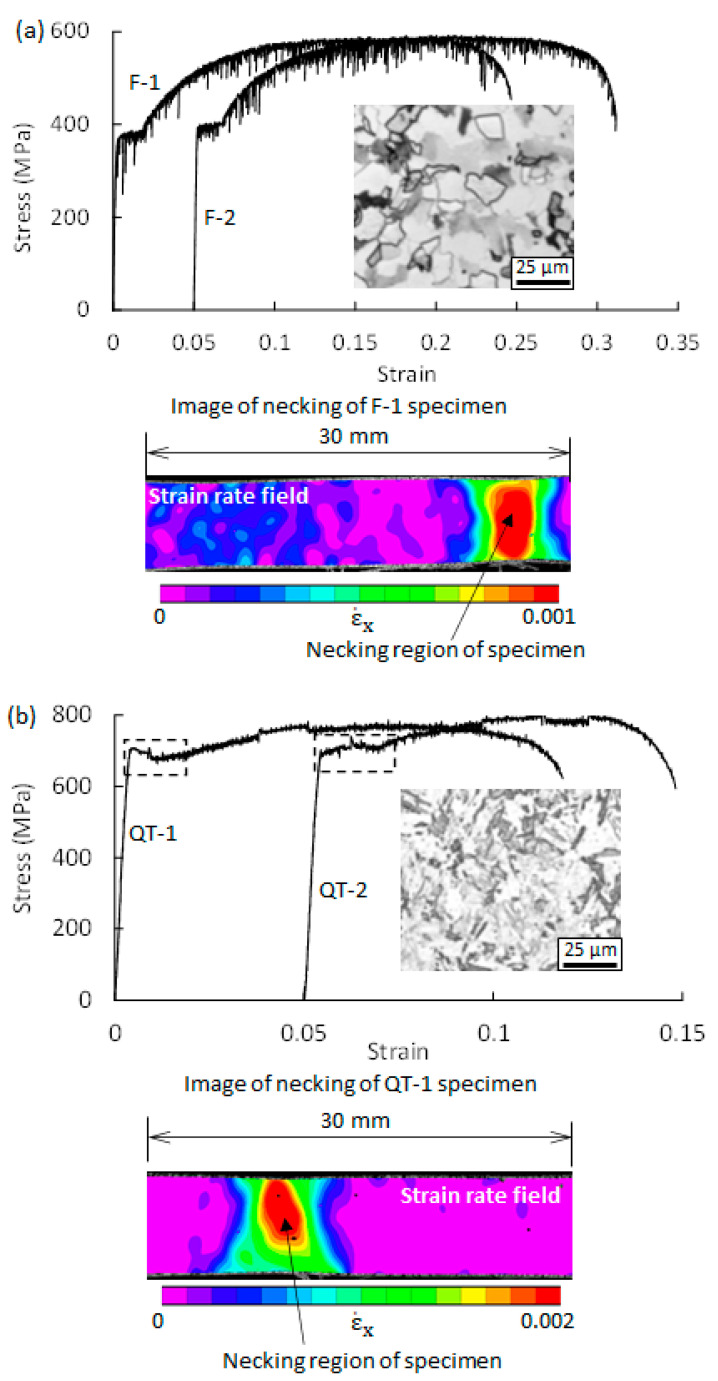
Macroscopic stress–strain curves of (**a**) ferrite/pearlite steel (F steel) and (**b**) tempered martensite steel (QT steel). Two tests were performed for each steel (F-1 and F-2 for F steel; QT-1 and QT-2 for QT steel).

Two tension tests were carried out for each steel. The obtained macroscopic stress–strain curves that show the average deformation behavior over a gauge length of 30 mm are provided in Figure 2. If the two stress–strain curves for one steel are plotted in the same coordinate system, the curves will be overlapped, and it is difficult to distinguish the individual curves. To clearly identify the individual curves of one steel, the two curves (F-2 and QT-2) were intentionally shifted 0.05 along the macroscopic strain axis. The optical microstructures of F steel and QT steel are shown in Figure 2.

The stress–strain curve of F steel shows a typical characteristic of Lüders deformation, i.e., a yield plateau. This means that the Lüders phenomenon inevitably occurs in F steel. In contrast to F steel, the stress–strain curve of QT steel does not show clear evidence of Lüders deformation and the PLC effect. This indicates that the occurrence of plastic instability in QT steel cannot be identified only from the stress–strain curve. Digital image correlation was used to analyze the plastic deformation behavior in the following section. It is noted that a region of the stress–strain curve of QT steel is enclosed with a dotted rectangle. It was found that plastic instability takes place in the rectangle, which will be described in detail later.

### 3.2. Plastic Instability

Lüders deformation or the PLC effect is characterized by the plastic band. Previous studies [4,5] showed that the strain-rate field could effectively identify the moving plastic bands. In the present study, the deformation process on the front surface of tension specimens was digitalized by a camera, and the obtained digital images over the whole tension process were used to analyze the strain and strain-rate field using two-dimensional DIC. The analysis via the strain-rate field for the F steel and QT steel shows that the plastic band occurs in two regions: (1) the elastic-to-plastic transition region (i.e., the Lüders phenomenon), and (2) the region after the onset of the necking of the tension specimen. This means that before the onset of the necking of the specimen, only the Lüders phenomenon exists in both steels. It is well known that the necking of the specimen induces plastic instability. This kind of plastic instability is not our concern, and it will not be discussed in the present study.

**Figure 3:**
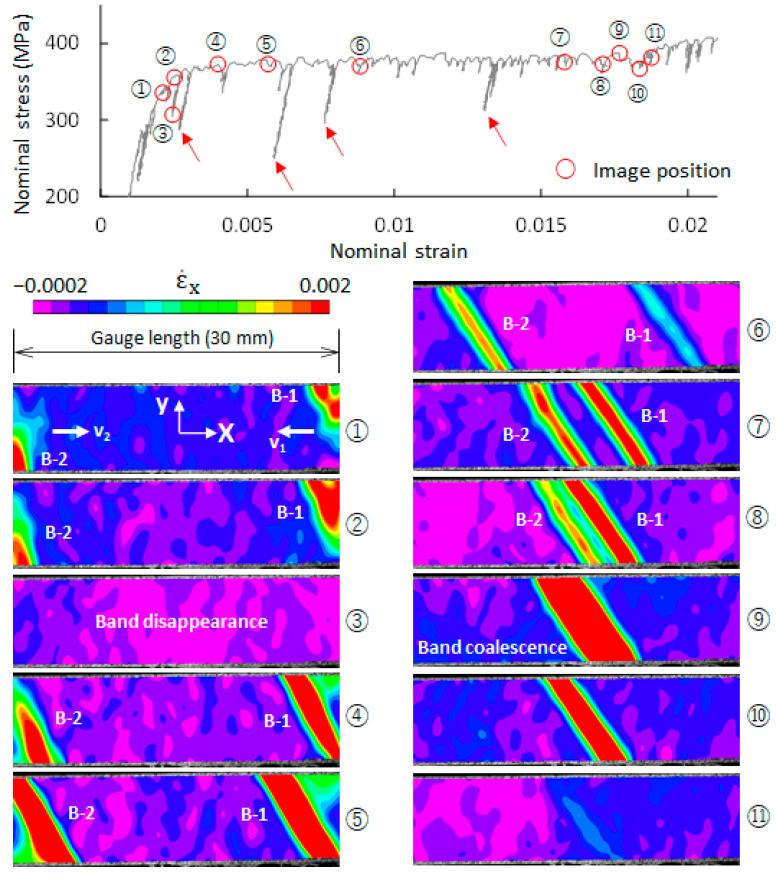
Evolution of plastic bands in ferrite/pearlite steel (F-1 specimen). Eleven images (① to ⑪) on the macroscopic stress–strain curve were taken. Their strain-rate fields over the gauge length (30 mm) show the evolution of plastic bands.

For the F steel, the Lüders phenomenon occurs mainly on the yield plateau. Eleven images (image ① to image ⑪) on the yield plateau of the F-1 specimen (cf. Figure 3) were selected, and their strain-rate fields over a gauge length of 30 mm are shown in Figure 3. It is generally believed that the pinning and the unpinning of dislocations cause the formation of a Lüders band. To form a Lüders band, a certain level of stress is required [35]. Apparently, at a given applied stress level, a site with a high-stress concentration more easily meets this critical stress condition than do other sites with low-stress concentrations. The shoulder of a specimen produces a high-stress concentration, providing an appropriate site for Lüders band nucleation as a result. As shown in image ①, two plastic bands (B-1 and B-2) formed near the right and left shoulders of the specimen. It can be seen that the position of image ① on the stress–strain curve is ahead of the yield point. This indicates that plastic bands were formed ahead of the yield point, which agrees with the observations of the previous study [4,5].

After band nucleation, B-1 propagates from right to left, while B-2 propagates toward the right. Lüders band propagation is characterized by the moving of a leading band front into the adjacent elastic region. It is also a repeated process of pinning and unpinning of dislocations. Naturally, the applied stress is required to exceed a critical stress level. Band propagation velocity was reported to be related to the magnitude of applied stress [4]. As shown in Figure 3, the applied stress on the yield plateau essentially remains constant, but it fluctuates significantly at some points on the yield plateau, and the applied stresses greatly decrease at those points. The applied stress corresponding to image ③ is too low to exceed the required critical stress for unlocking the dislocations, resulting in band disappearance. The strain-rate field of image ③ verifies the disappearance of the two plastic bands. When the applied stress recovers to its original value, the two bands appear again at the original sites. In Figure 3, four arrows show four low-stress levels. The two bands also disappear around the four stress levels. B-1 and B-2 propagate oppositely until coalescing with each other (cf. image ⑨). The position of image ⑪ is nearly the end of the yield plateau, and the coalesced band almost disappears at this point.

**Figure 4:**
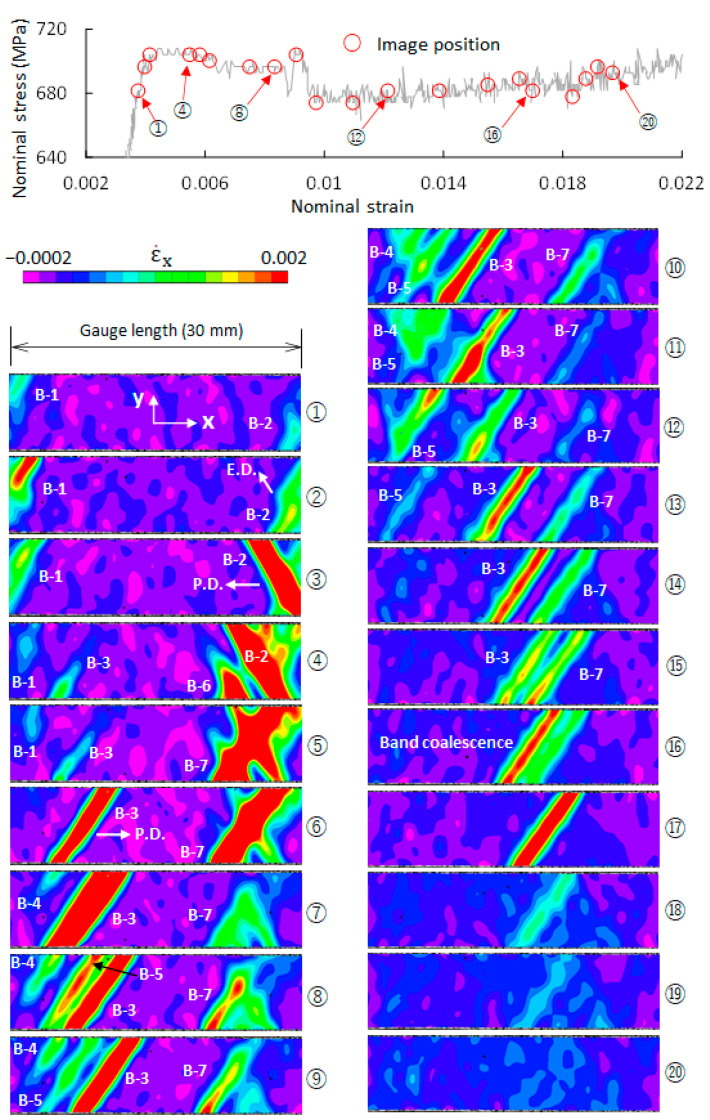
Evolution of plastic bands in tempered martensite steel (QT-1 specimen). Twenty images (① to ⑳) on the macroscopic stress–strain curve were taken. Their strain-rate fields over the gauge length (30 mm) show the evolution of plastic bands. B-1 to B-7, band 1 to band 7; E. D., extension direction; P. D., propagation direction.

The deformation process of QT steel over the whole stress–strain curve was examined via DIC. It was found that plastic instability occurred only within a certain range of the stress–strain curve. The range is enclosed in Figure 2 by a dotted rectangle. The QT-1 specimen was taken to show in detail the evolution of the plastic instability in QT steel. Twenty typical points on the macroscopic stress–strain curve of the QT-1 specimen were selected (cf. Figure 4). The strain-rate fields corresponding to the 20 points (image ① to image ⑳) are given in Figure 4. It can be seen that two bands (B-1 and B-2) formed near the left and right shoulders of the specimen in image ①, respectively. The position of image ① in the stress–strain curve indicates that a band nucleated ahead of the yield point. The variation in the value of the strain rate within B-1 from image ① to image ⑥ shows the evolution of B-1, in that the band first grows (①→②), and then decays, and finally completely disappears (②→⑥). The position of B-1 in images ①→⑤ is almost unchanged. This indicates that the band formation, growth, and disappearance of B-1 took place at almost the same site, and apparently, band propagation did not occur.

After band formation, B-2 grows along one direction from image ① to image ②and then extends along another direction, shown by an arrow in image ②, instead of in its original direction. Image ③ shows the appearance of B-2 after the change in the direction of extension. B-2 propagates from right to left from image ③ to image ④. In image ④, a new band (B-6) on the left side of B-2 was formed. B-6 and B-2 coalesced and extended across the width of the specimen to produce a new band with several branches (B-7) in image ⑤. The position of B-7 is almost unchanged from image ⑤ to image ⑮. This indicates that B-7 did not propagate after band formation.

A band (B-3) was formed in image ④, which is after the upper yield point. This band extends across the width of the specimen from image ④ to image ⑥ and then propagates from left to right. In image ⑧, a new band (B-5) was split out of B-3, and B-3 continues to propagate until coalescing with B-7 in image ⑯. The coalesced band gradually decays and finally disappears. The split band (B-5) gradually decays without band propagation and finally disappears in image ⑭. B-4 experiences a process of band formation, growth, and disappearance from image ⑦ to image ⑬ that is similar to B-1 and B-5. It can be seen from Figure 3 and Figure 4 that the Lüders deformation process in QT steel is more complicated than that in F steel.

**Figure 5:**
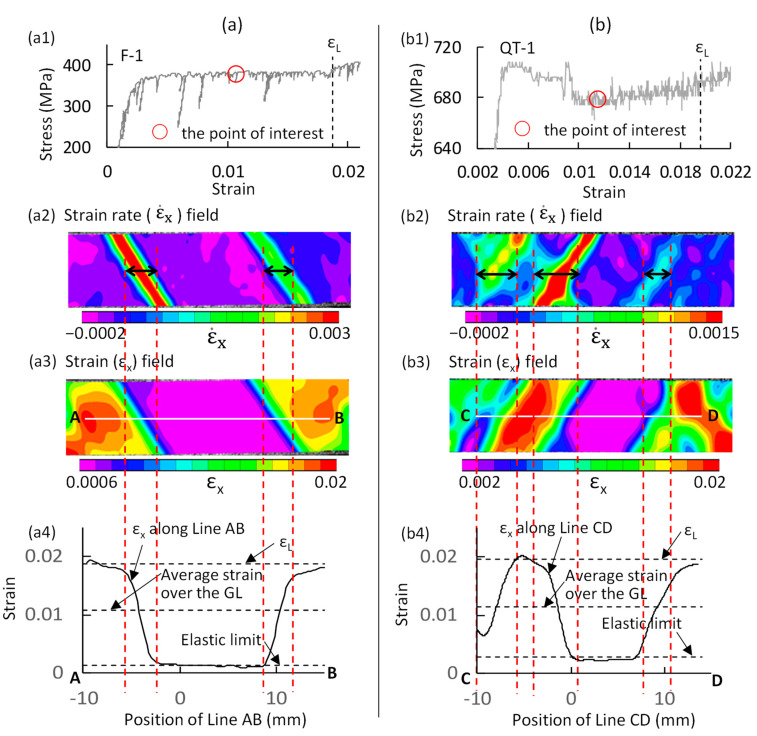
Local strain (ε_x_) distributions in (**a**) F steel (F-1 specimen) and (**b**) QT steel (QT-1 specimen) over the gauge length at a point on the macroscopic stress–strain curve located at almost the middle of the Lüders deformation process. The strain-rate field shows the status of the moving bands, and the strain field shows the two-dimensional strain distribution at the point of interest. The local strain (ε_x_) distributions along the AB and CD lines were extracted. The average strain over the GL is the macroscopic strain of the stress–strain curve corresponding to the point of interest. ε_L_, Lüders strain.

Because Lüders deformation occurs in the elastic-to-plastic transition region, the plastic region and the elastic region are simultaneously present in the Lüders deformation process. This indicates that the strain distribution over the specimen is heterogeneous. In Figure 5, one point on the stress–strain curve of (a) F steel (F-1 specimen) and (b) QT steel (QT-1 specimen) that is nearly in the middle of Lüders deformation process is selected. The strain heterogeneity in the two steels is revealed by analyzing the strain distribution at this point. The experimental data of the F-1 specimen and the QT-1 specimen at this point are summarized in columns (a) and (b), respectively.

The strain-rate (${\dot{\varepsilon }}_{x}$) field of the F-1 specimen (cf. Figure 5(a2)) shows that two moving plastic bands exist. The corresponding strain ($\varepsilon_{x}$) field was described in terms of two-dimensional contour (Figure 5(a3)). The strain field is roughly divided into three regions: the middle is the elastic region, and the others are plastic regions. To quantitatively describe the strain distribution, a center line (line AB) is drawn in the two-dimensional strain contour, and the strain ($\varepsilon_{x}$) along the line is extracted and shown in Figure 5(a4). Three reference points, which were directly derived from the macroscopic stress–strain curve, are also provided: the Lüders strain ($\varepsilon_{L}$), the average strain over the gauge length (GL), and the elastic limit. The Lüders deformation process is characterized by the formation of Lüders bands (single or multiple bands), followed by band propagation over the whole specimen. The strain corresponding to the ending point of the Lüders deformation process is referred to as the Lüders strain. For the steel with a clear yield plateau, the ending point of the Lüders deformation process is generally around the ending point of the yield plateau. This point in F-1 and QT-1 specimens were directly determined by the strain-rate field in the present study. The average strain over the GL is the macroscopic strain of the stress–strain curve of the F-1 specimen corresponding to the point of interest. Careful examination of the macroscopic stress–strain curve in the macroscopic elastic region (i.e., from point zero to the upper yield point) shows that stress linearly increases with strain only within the initial region (from point zero to a certain stress level); beyond this region, stress gradually deviates from this straight line. The maximum macroscopic strain of the linear range is denoted as the elastic limit.

Figure 5(a4) shows that the middle region is in the elastic state, and the corresponding strain is almost equal to the elastic limit. The width of the two moving plastic bands with respect to Line AB is marked by an arrow (↔) in Figure 5(a2). The range of Line AB enclosed by the band width is shown by two vertical dotted lines in red in Figure 5(a3,a4). Figure 5(a4) shows that the strain varies significantly within the band width from an elastic strain to a large plastic strain (close to the $\varepsilon_{L}$). The average strain over the GL is the average value of the elastic and plastic regions.

The strain heterogeneity in the QT-1 specimen was analyzed in a similar way in Figure 5b. As shown in Figure 5(b2), the shape of the moving bands is more complicated than that in the F-1 specimen. The local strain along Line CD (cf. Figure 5(b3)) is shown in Figure 5(b4). The strain in the elastic region (middle region) is close to the elastic limit. The width of three moving bands with respect to Line CD is shown by an arrow (↔) in Figure 5(b2). The strain variation within the band width is similar to that in the F-1 specimen. The maximum plastic strain within the band width also approaches the $\varepsilon_{L}$. The strain distribution in the QT-1 specimen is more complicated than that in the F-1 specimen.

## 4. Conclusions

The plastic deformation behavior of medium-carbon tempered martensite steel at room temperature was investigated. The results obtained regarding the plasticity of tempered martensite were as follows.

(1) In the elastic-to-plastic transition region, there is no clear yield plateau on the stress–strain curve of medium-carbon tempered martensite steel;
(2) The Lüders deformation phenomenon is present, but the Portevin-Le Châtelier phenomenon is not found;
(3) The elastic and plastic regions are simultaneously present in the Lüders deformation process. The local strain in the elastic region is close to the elastic limit. The variation in strain within a Lüders band is significant, monotonously increasing from an elastic strain to a large plastic strain that is close to the Lüders strain;
(4) The local strain distribution in tempered martensite steel during the Lüders deformation process is more complicated than that in ferrite steel.

## Data Availability

Not applicable.

## References

[1] Piobert A. (1842). Expériences sur la pénétration des projectiles dans le fer forgé. Mémoire L’artillerie.

[2] Lüders W. (1860). Ueber die aeuerung der elasticität an stahlartigen eisenstäben und stahlstäben, und über eine beim biegen solcher stäbe beobachtete molecularbewegung. Dingler Polytech. J..

[3] Portevin A., le Châtelier F. (1923). Sur un phénomène observé lors de l′essai de traction d′alliages en cours de transformation. Comptes Rendus Ácadémie Sci. Paris.

[4] Qiu H., Inoue T., Ueji R. (2020). Experimental measurement of the variables of Lüders deformation in hot-rolled steel via digital image correlation. Mater. Sci. Eng. A.

[5] Qiu H., Inoue T., Ueji R. (2020). In-Situ observation of Lüders band formation in hot-rolled steel via digital image correlation. Metals.

[6] Kozłowska A., Grzegorczyk B., Morawiec M., Grajcar A. (2019). Explanation of the PLC effect in advanced high-strength medium-Mn steels. Materials.

[7] Renard K., Ryelandt S., Jacques P.J. (2010). Characterisation of the Portevin-Le Châtelier effect affecting an austenitic TWIP steel based on digital image correlation. Mater. Sci. Eng. A.

[8] Sarmah R., Ananthakrishna G. (2015). Correlation between band propagation property and the nature of serrations in the Portevin–Le Chatelier effect. Acta Mater..

[9] Cai Y., Zhang Q., Yang S., Fu S., Wang Y. (2016). Experimental study on three-dimensional deformation field of Portevin–Le Chatelier effect using digital image correlation. Exp. Mech..

[10] Penning P. (1972). Mathematics of the Portevin-Le Châtelier effect. Acta Metall..

[11] Jiang H.F., Zhang Q.C., Chen X.D., Chen Z.J., Jiang Z.Y., Wu X.P., Fan J.H. (2007). Three types of Portevin–Le Chatelier effects: Experiment and modelling. Acta Mater..

[12] Lloyd D.J., Morris L.R. (1977). Lüders band deformation in a fine grained aluminium alloy. Acta Metall..

[13] Delwiche D.E., Moon D.W. (1972). Strain profile of a propagating Lüders front. Mater. Sci. Eng..

[14] van Rooyen G.T. (1971). Basic factors which influence the Lüders strain during discontinuous yielding. Mater. Sci. Eng..

[15] Tsuchida N., Masuda H., Harada Y., Fukaura K., Tomota Y., Nagai K. (2008). Effect of ferrite grain size on tensile deformation behavior of a ferrite-cementite low carbon steel. Mater. Sci. Eng. A.

[16] Tsuchida N., Tomota Y., Nagai K., Fukaura K. (2006). A simple relationship between Lüders elongation and work-hardening rate at lower yield stress. Scr. Mater..

[17] El-Magd E., Scholles H., Weisshaupt H. (1996). Influence of strain rate on the stress–strain curve in the range of Lüders strain. Steel Res..

[18] van Rooyen G.T. (1968). The stress and strain distribution in a propagation Lüders front accompanying the yield-point phenomenon in iron. Mater. Sci. Eng..

[19] Cai Y.L., Yang S.L., Fu S.H., Zhang Q.C. (2016). The inﬂuence of specimen thickness on the Lüders effect of a 5456 Al-based alloy: Experimental observations. Metals.

[20] Zhang M.H., Li L.F., Ding J., Wu Q.B., Wang Y.D., Almer J., Guo F.M., Ren Y. (2017). Temperature-dependent micromechanical behavior of medium-Mn transformation-induced-plasticity steel studied by in situ synchrotron X-ray diffraction. Acta Mater..

[21] Grzegorczyk B., Kozłowska A., Morawiec M., Muszyński R., Grajcar A. (2019). Effect of deformation temperature on the Portevin-Le Chatelier effect in medium-Mn steel. Metals.

[22] Lee S.W., Estrin Y., de Cooman B.C. (2014). Effect of the strain rate on the TRIP–TWIP transition in austenitic Fe-12 pct Mn-0.6 pct C TWIP steel. Metal. Mater. Trans. A.

[23] Cottrell A.H., Bilby B.A. (1949). Dislocation theory of yielding and strain ageing of iron. Phys. Soc..

[24] Cottrell A.H. (1953). A note on the Portevin-le Chatelier effect. Lond. Edinb. Dublin Philos. Mag. J. Sci..

[25] Marais A., Mazière M., Forest S., Parrot A., le Delliou P. (2012). Identification of a strain-aging model accounting for Lüders behavior in a C-Mn steel. Philos. Mag..

[26] Wang X.G., Wang L., Huang M.X. (2017). Kinematic and thermal characteristics of Lüders and Portevin-Le Chatelier bands in a medium Mn transformation-induced plasticity steel. Acta Mater..

[27] Tsukahara H., Iung T. (1998). Finite element simulation of the Piobert–Lüders behavior in an uniaxial tensile test. Mater. Sci. Eng. A.

[28] McCormick P.G. (1988). Theory of flow localization due to dynamic strain aging. Acta Metall..

[29] Hähner P. (1993). Modelling the spatiotemporal aspects of the Portevin-Le Chatelier effect. Mater. Sci. Eng. A.

[30] Fressengeas C., Beaudoin A.J., Lebyodkin M., Kubin L.P., Estrin Y. (2005). Dynamic strain aging: A coupled dislocation—Solute dynamic model. Mater. Sci. Eng. A.

[31] Onodera R., Nonomura M., Aramaki M. (2000). Stress drop, Lüders strain and strain rate during serrated flow. J. Jpn. Inst. Met..

[32] Hahn G.T. (1962). A model for yielding with special reference to the yield-point phenomena of iron and related BCC metals. Acta Met..

[33] Liu C.Q., Peng Q.C., Xue Z.L., Deng M.M., Wang S.J., Yang C.W. (2018). Microstructure-tensile properties relationship and austenite stability of a Nb-Mo micro-alloyed medium-Mn TRIP steel. Metals.

[34] Sun B.H., Vanderesse N., Fazeli F., Scott C., Chen J.Q., Bocher P., Jahazi M., Yue S. (2017). Discontinuous strain-induced martensite transformation related to the Portevin-Le Chatelier effect in a medium manganese steel. Scr. Mater..

[35] Schwab R., Ruff V. (2013). On the nature of the yield point phenomenon. Acta Mater..

